# Beyond Numbers: How Biochemical Parameters Can Predict Outcomes in Chronic Kidney Disease Patients on Maintenance Hemodialysis

**DOI:** 10.7759/cureus.67349

**Published:** 2024-08-20

**Authors:** Imran A Siddiqui, Afshan Masood, Sushmita Chandagiri, Raichur v Kumar, Altaf A Mir

**Affiliations:** 1 Biochemistry, ESIC Medical College and Superspeciality Hospital, Sanathnagar, Hyderabad, IND; 2 Biochemistry, Obesity Research Centre, College of Medicine, King Saud University, Riyadh, SAU; 3 Nephrology, ESIC Medical College and Superspeciality Hospital, Sanathnagar, Hyderabad, IND; 4 Biochemistry, All India Institute of Medical Sciences, Raebareli, IND

**Keywords:** biochemical evaluation, esrd, magnesium, homocysteine, ckmbd, biochemical markers, ckd

## Abstract

Introduction

The treatment and management of patients undergoing maintenance hemodialysis (MHD) requires constant evaluation through the assessment of biochemical markers. This is necessary for treatment, to prevent progression to complications such as mineral bone disease, and to improve quality of life. We aimed to study the biochemical profile of patients with chronic kidney disease (CKD) grades 4 and 5 on MHD, identify markers altered due to different etiologies, duration of illness, and duration of hemodialysis, and create a panel of markers that can be useful in planning better management.

Methods

All consecutive patients attending the dialysis unit of ESIC Super Speciality Hospital with CKD grade 4 or grade 5 on MHD between 2019 and 2020 were recruited. A detailed clinical history and demographic profile were taken, and blood samples were collected from the patients during follow-up visits in plain and EDTA (ethylenediamine tetraacetic acid) tubes for analysis.

Results

A total of 312 patients (22.1% females and 77.9% males.) with a mean age of 49.74 ± 11.49 years were recruited. In the study population, diabetic nephropathy (DN) (17%) and hypertensive nephropathy (48.7%) were the two most prevalent causes of CKD. The majority (64%) of the patients were on MHD three times a week. The range of estimated glomerular filtration rate (eGFR) (ml/min/1.73 m^2^) at the time of initiation of MHD was 2.9-26.8 according to the Chronic Kidney Disease Epidemiology Collaboration (CKD-EPI) formula. The mean duration of MHD was 51.58 months, with a mortality rate of 5.9% during the follow-up period (3-108 months).

Conclusion

Optimal selection and combination of biochemical tests will help in ascertaining the adequacy of management, progress of disease, or complications in MHD patients. This in turn will help guide the clinicians in effectively using these markers in their day-to-day practice.

## Introduction

Chronic kidney disease (CKD) is considered a major noncommunicable disease along with diabetes mellitus (DM) and hypertension (HTN), adding significantly to the global burden of disease. Regardless of their etiology, abnormalities of kidney function or structure presenting for a duration of more than 3 months and resulting in a progressive decline of renal function are commonly grouped together as CKD. The underlying pathology is either a systemic disease such as diabetes and hypertension leading to nephropathy or non-systemic pathophysiologic processes that include glomerulonephritis, polycystic kidney disease, nephrolithiasis, prostate disease, or malignancy.

The global prevalence of CKD is 9.1%, with India contributing a dramatic 17%. The increased prevalence of CKD is paralleled by an increased incidence of end-stage renal disease (ESRD) and a rise in mortality, making it the ninth leading cause of death in the Indian population [1-3]. The rising mortality rates are attributed to the delayed diagnosis, as no marker to date effectively predicts the onset of CKD or its progression to ESRD. CKD is usually detected during routine laboratory evaluation of kidney disease by measuring serum creatinine levels, a decline in estimated glomerular filtration rate (eGFR), or proteinuria. A progressive decline in kidney function and a decreasing eGFR in ESRD require renal replacement therapy (RRT), which is necessary to sustain and continue life [4, 5].

The most accepted form of RRT is hemodialysis, which has become the mainstay of RRT worldwide and in India, accounting for 80% of patients in the USA [6]. In India, over 130,000 patients receive dialysis, and the number is increasing by about 232 per million, a reflection of increasing longevity in general [7]. However, data from large cohorts of the Indian population is unavailable. The management and evaluation of patients on maintenance hemodialysis are critical as they allow them to have better clinical care, preventing disease worsening and the development of complications. Developing accurate biomarkers for evaluating ESRD progression or predicting and preventing complications constitutes a clinical challenge.

The clinical chemistry laboratory is vital in accurately diagnosing kidney disease and advancing kidney dysfunction. Still, no panels exist to predict the progression or the risk of developing complications. Recent studies have identified proteins, such as FGS23, that can be biomarkers for diagnosing CKD and ESRD. However, they remain mainly as research molecules and are rarely implemented in chronic patient management. Further grading of CKD relies solely on estimating the eGFR and assessing albuminuria (proteinuria). Higher serum creatinine levels, or proteinuria, are incidental findings that lead to a diagnosis of CKD.

Serum creatinine levels are found to be normal despite the loss of more than 50% of renal function, which decreases the sensitivity of these markers in detecting disease progress as the breaking point is different in every patient. This makes it pertinent to effectively use the other available tests in the clinical laboratory, such as vitamin D, alkaline phosphatase (ALP), calcium, phosphorus, parathyroid hormone, serum electrolytes, and magnesium, for diagnosis and prognosis. Hence, multimarker panels targeting progress or complications, rather than undirected testing, will help with better clinical decisions and management.

Gaining insight into the variations of these parameters in serum levels could aid in the noninvasive differentiation of kidney diseases, tracking treatment efficacy, and forecasting the likelihood of progression to severe outcomes and associated complications. This could support the prudent utilisation of these tests for informed clinical decision-making. Therefore, our study aims to pinpoint the biochemical parameters that accurately reflect notable alterations in the clinical status of individuals with CKD receiving MHD.

## Materials and methods

The study protocol was approved by the Ethics Committee of the ESIC Medical College and Hospital (approval number: 799/U/IEC/ESICMC/F0186/06-2019). Consecutive patients followed up in the dialysis unit of ESIC Super Speciality Hospital, Sanathnagar, Hyderabad, with CKD grades 4 and 5 on MHD, were enrolled prospectively over a period of one year, i.e., from July 2019 to June 2020. After approval, informed consent was obtained from all the patients. The patient and their relatives were subsequently interviewed, and the data were entered into an electronically compatible proforma.

Patients were diagnosed with CKD grades 4 and 5 if they had an irreversible decline in renal function for more than three months [8, 9]. The diagnosis of underlying kidney disease was based on clinical, laboratory, and radiological features. The serum creatinine was more than 1.5 mg/dL, and the urea was more than 40 mg/dL for more than three months [10-11]. Clinical and anthropometric data were collected on the day of blood sampling: age, sex, weight, height, previous medical history, and concomitant treatment were noted. For routine analysis, 5 mL of venous blood was drawn. All blood samples were drawn at least 16 days after each MHD patient's last intravenous (IV) iron administration.

The laboratory values were measured immediately before the dialysis session. Serum hs-CRP (high-sensitivity C-reactive protein) was calculated as an indicator of an inflammatory state and assessed by an immuno-turbidimetric method. Laboratory measurements were performed by Roche Cobas Integra 400 plus (Roche Diagnostics Corp., Indianapolis, USA), and the hormonal parameters were evaluated in Roche Cobas e411 (Roche Diagnostics Corp., Indianapolis, UA). Laboratory tests included haemoglobin, serum creatinine, urea, uric acid, glucose, total cholesterol, triglycerides, alkaline phosphatase (ALP), phosphorus, calcium, albumin, magnesium, iron, ferritin, parathyroid hormone (PTH), vitamin D, hs-CRP, homocysteine, ionised calcium, bicarbonates, and electrolytes. Calculated values were obtained for blod urea nitrogen (BUN (mg/dL) = Urea (mg/dL) / 2.1428) and eGFR using the CKD-EPI formula.

All patients with anaemia, neoplasm, connective tissue disease, infection, allergy, treatment with potentially nephrotoxic drugs, and with known renal disease other than CKD were excluded. Moreover, patients with poorly controlled hypertension, decompensated heart failure, urinary tract infections after increased physical activity, women during menstruation, and pregnant women were excluded to avoid nonspecific albuminuria.

Data were presented as several patients (percentages) for categories, mean ± standard deviation, or median (lower-upper quartile) for quantitative variables with normal and non-normal distributions (as checked using Shapiro-Wilk’s test). Spearman’s rank correlation coefficient was used to study correlations. Differences between groups were tested with a t-test and an ANOVA test. Simple and multiple logistic regressions were used to assess predictors of CKD. Odds ratios (OR) with 95% confidence intervals (95% CI) were reported as logistic regression results. The variables that proved significant predictors in simple logistic regression were studied further using receiver operating characteristic (ROC) curve analysis to assess the best cut-off, sensitivity, and specificity. The statistical tests were two-tailed, and the results were considered significant at a p-value of less than 0.05. All statistical analyses were performed with SPSS version 24.0 (IBM Corp., Armonk, USA).

## Results

The mean age of the study population was 49.74 ± 11.49 years, wherein 22.1% (n = 69) were females and 77.9% (n = 243) were males. The mean duration of CKD was observed to be 72.39 ± 51.83 months, and the mean duration of hemodialysis in our study group was observed to be 51.58 ± 33.78 months. The baseline characteristics of the patients are shown in Table 1. 

**Table 1:** Baseline characteristics of patients receiving maintenance hemodialysis patients (n = 312): eGFR: estimated glomerular filtration rate, PTH: parathyroid hormone, MHD: maintenance hemodialysis, ALP: alkaline phosphatase, hs-CRP: High-sensitivity C-reactive protein, CKD – EPI: Chronic Kidney Disease Epidemiology Collaboration, CKD: Chronic Kidney Disease, HTN: hypertension,

Characteristics	n = 312
	Mean ± SD, N (%)
Age (years)	49.74 ± 11.49
Gender (Male), n (%)	243(77.9%)
(Female), n (%)	69(22.1)
Mean duration of CKD (months)	72.39 ± 51.83
Mean duration of MHD (months)	51.58 ± 33.78
Diabetes Mellitus, n (%)	53 (17)
HTN, n (%)	152 (48.7)
Urea (mg/dL)	76.2 ± 24.19
Creatinine (mg/dL)	7.3 ± 2.36
eGFR (CKD – EPI) (mL/min/1.73m^2^)	8.59 ± 3.87
Potassium (mmol/L)	4.68 ± 0.69
Calcium (mg/dL)	8.99 ± 0.81
hs-CRP (mg/dl)	1.32 ± 2.17
Homocysteine (umol/L)	24.54 ± 17.3
Phosphorous (mg/dL)	4.91 ± 1.43
ALP (IU/L)	219.48 ± 183.9
PTH (pg/mL)	638.39 ± 410.02
Vitamin D (ng/mL)	42.27 ± 20.2
Albumin (g/dL)	4.1 ± 0.47
Ferritin (ug/L)	950.35 ± 711.18

In the overall MHD group (Table 1) we found that serum levels of urea (76.2 ± 24.19) mg/dL, creatinine (7.3 ± 2.36) mg/dL, PTH (638.39 ± 410.02) pg/mL, ferritin (950.35 ± 711.18) ug/L, hs-CRP (1.32 ± 2.17) mg/dL, and homocysteine (24.54 ± 17.3) umol/L were increased when compared to the normal reference ranges in all the study population whereas mean eGFR was reduced (8.59 ± 3.87 ml/min/1.73m^2^ ). 

The patients undergoing MHD were stratified and evaluated by classifying them based on the median duration of CKD (62 months), followed by the median duration of maintenance hemodialysis (43 months), presence or absence of systemic disease, based on the etiology of systemic disease (diabetes and hypertension) and lastly based on survival vs non-survival.

When the patients were grouped based on the duration of CKD of more than 62 months, it was observed that mean ALP IU/L (266.5 ± 225.93 vs 170.6 ± 106.88 p <0.001), Vitamin D ng/mL (48.62 ± 19.37 vs 35.81 ± 19.22 p<0.001) and iron ug/dL (95.6 ± 46.5 vs 83.36 ± 41.13, p =0.02), was significantly more in the group with longer duration of disease (p <0.05). Mean homocysteine umol/L (27.33 ± 21.37 vs 21.88 ± 11.75 p=0.025) was significantly lower in the group with a longer duration of disease (Table 2).

**Table 2:** Changes in the levels of the measured parameters with duration of disease: BUN: blood urea nitrogen, iCa: ionised calcium, PTH: parathyroid hormone, ALP: alkaline phosphatase, eGFR: estimated glomerular filtration rate, CKD: chronic kidney disease, Ca: calcium, Ca x P: calcium-phosphate product

	CKD Duration: ≤ 61 Months	CKD Duration: ≥ 62 Months	p-value	MHD Duration: ≤ 43 Months	MHD Duration: ≥ 43 Months	p-value
	Mean ± SD	Mean ± SD		Mean ± SD	Mean ± SD	
Age (years)	48.75 ± 11.25	50.69 ± 11.67	.137	48.19±12	51.38±10.72	.014
Urea (mg/dL)	78.56 ± 25.15	73.96 ± 23.11	.093	77.5±24.78	74.83±23.56	.330
Creatinine (mg/dL)	7.36 ± 2.34	7.24 ± 2.39	.648	7.37±2.39	7.23±2.34	.607
BUN (mg/dL)	36.37 ± 11.64	34.24 ± 10.7	.093	35.88±11.47	34.64±10.91	.332
Sodium (mmol/L)	139.5 ± 4.02	139.54 ± 3.98	.934	139.14±4.26	139.91±3.66	.088
Potassium (mmol/L)	4.73 ± 0.76	4.63 ± 0.62	.220	4.72±0.75	4.64±0.62	.299
iCa (mmol/L)	0.99 ± 0.12	1.01 ± 0.12	.194	0.99±0.12	1±0.12	.532
Calcium (mg/dL)	8.97 ± 0.85	9.01 ± 0.78	.637	9.05±0.8	8.93±0.82	.163
Calcium adjusted by albumin (mg/dL)	8.86 ± 0.85	8.96 ± 0.79	.296	8.95±0.82	8.86±0.82	.344
Phosphorous (mg/dL)	5.05 ± 1.4	4.77 ± 1.46	.084	5.02±1.43	4.79±1.44	.167
Bicarbonate (mmol/L)	19.56 ± 3.75	19.31 ± 3.46	.532	19.3±3.88	19.57±3.29	.516
PTH (pg/mL)	596.42 ± 392.6	678.26 ± 423.28	.078	581.41±381.91	698.37±430.82	.012
Vitamn D (ng/mL)	35.81 ± 19.22	48.62 ± 19.37	<0.001	36.79±19.02	47.47±20.18	<0.001
Iron (ug/dL)	83.36 ± 41.13	95.6 ± 46.5	.020	87.13±43.92	92.21±44.73	.335
Ferritin (ug/L)	866.91 ± 668.5	1025.8 ± 741.88	.063	862.63±681.26	1036.82±731.64	.041
Magnesium (mg/dL)	2.49 ± 0.41	2.43 ± 0.33	.206	2.52±0.4	2.4±0.33	.019
Albumin (g/dL)	4.14 ± 0.44	4.07 ± 0.51	.211	4.13±0.47	4.08±0.49	.346
ALP (IU/L)	170.6 ± 106.88	266.5 ± 225.93	<0.001	160.23±102.59	282.68±226.1	<0.001
Homocysteine (umol/L)	27.33 ± 21.37	21.88 ± 11.75	.025	27.7±21.34	21.53±11.64	.011
eGFR	8.5 ± 3.78	8.67 ± 3.96	.701	8.58±3.83	8.59±3.92	.980
Duration CKD (months)	35.33 ± 12.82	109.71 ± 49.49	<0.001	45.22±34.65	101.67±51.41	<0.001
Duration MHD (months)	29.23 ± 14.62	73.08 ± 33.02	<0.001	25.12±11.2	79.1±26.64	<0.001
Ca x P (mg^2^/dL^2^)	45.46 ± 13.22	43.23 ± 14.03	.156	45.65±13.8	42.91±13.42	.081

A significant positive correlation was observed between the duration of CKD and PTH (r=0.228, p<0.001), vitamin D (r = 0.277, p <0.001), iron (r = 0.136, p = 0.026), and ALP (r = 0.219, p <0.001).

On the other hand, we found that mean age (years) (51.38±10.72 vs 48.19±12, p =0.014), PTH (pg/mL) (698.37±430.82 Vs 581.41±381.91, p = 0.012), vitamin D (ng/mL) (47.47±20.18 vs 36.79±19.02 p< 0.001), ferritin (ug/L) (1036.82±731.64 vs 862.63±681.26, p= 0.04), and ALP (IU/L) (282.68±226.1 vs 160.23±102.59 p<0.001) were significantly increased while mean serum magnesium (mg/dL) (2.4±0.33 vs 2.52±0.4, p= 0.019) and homocysteine (umol/L) (21.53±11.64 vs 27.7±21.34, p =0.011) levels were significantly less in patients with duration of MHD more than 43 months, p <0.05 (Table 2). 

When the patients were grouped according to their etiology, we observed that mean serum urea (mg/dL) (78.86 ± 25.22 vs 69.65±20.11, p = 0.002) and potassium (mmol/L) (4.75±0.71 vs 4.51±0.6, p = 0.004) levels were significantly higher in those with systemic disease compared to non-systemic disease (p <0.05) (Table 3).

**Table 3:** Changes in the levels of the measured parameters with presence or absence of systemic disease and with different etiologies (Diabetes or Hypertension): BUN: blood urea nitrogen, K: potassium, CKD: chronic kidney disease, eGFR: estimated glomerular filtration rate, HTN: hypertension

	Systemic Disease	Non-Systemic Disease	p-value	Diabetes	Hypertension	p-value
	Mean ± SD	Mean ± SD		Mean ± SD	Mean ± SD	
Age (years)	50.62±11.22	47.59±11.9	.035	56.64±7.92	49.16±11.22	.000
Urea (mg/dL)	78.86±25.22	69.65±20.11	.002	84.63±28.06	76.85±23.72	.052
Creatinine (mg/dL)	7.43±2.36	6.99±2.35	.135	6.8±2.42	7.74±2.31	.013
BUN (mg/dL)	36.51±11.68	32.24±9.31	.002	39.17±13	35.58±10.98	.052
K (mmol/L)	4.75±0.71	4.51±0.6	.004	4.78±0.68	4.75±0.73	.757
Vitamin D (ng/mL)	41.93±20.6	43.2±19.59	.679	35.08±18.24	43.56±21.04	.026
Albumin g/dL	4.09±0.49	4.12±0.46	.606	3.97±0.5	4.15±0.47	.025
eGFR (mL/min/1.73m^2^)	8.34±3.77	9.2±4.06	.076	9.31±5.06	7.9±3.17	.019
Duration of CKD (months)	70.92±49.74	76.21±57.04	.434	50.84±27.28	73.43±51.12	.004
Duration HTN (months)	51.48±34.41	51.84±32.29	.934	42.47±27.09	53.93±36.62	.038

Among the systemic diseases, diabetes and hypertension were compared. The markers that stood out were mean albumin (g/dL) (4.15±0.47 vs 3.97±0.5, p 0.025), creatinine (mg/dL) (7.74 ± 2.31 vs 6.8 ± 2.42 p = 0.013), vitamin D (ng/mL) (43.56 ± 21.04 vs 35.08 ± 18.24, p = 0.026) levels, which were significantly higher in patients with hypertension whereas mean age and eGFR were significantly lower in patients with hypertension compared to diabetes (p <0.05) (Table 3).

On evaluating the patients with Chronic Kidney Disease - Mineral Bone Disorder (CKD-MBD)** **and non-CKD-MBD, mean urea mg/dl (87.22±26.47 vs 73.07±22.6 p <0.001), creatinine (mg/dL) (8.53±2.39 vs 6.95±2.24 p <0.001), ALP (IU/L) (330.88±217 vs 187.58±160.05 p <0.001), phosphorus (mg/dL) (5.81±1.4 vs 4.65±1.34 p <0.001), the product of calcium and phosphorus (mg^2^/dL^2^) (50.34±14.08 vs 42.54±13.04 p <0.001), PTH (pg/mL) (788.43±485.89 vs 595.79±376.12, p =0.001) and mean duration of MHD (months) (58.89±30.81 vs 49.57±34.33 p = 0.047), were significantly higher in patients with CKD-MBD whereas mean ionized calcium (mmol/L) (0.96±0.12 vs 1.01±0.12 p = 0.002), serum calcium (mg/dL) (8.61±0.96 vs 9.1±0.73 p <0.001), corrected calcium (mg/dL) (8.52±0.93 vs 9.02±0.75 p <0.001), bicarbonate (mmol/L) (18.61±3.86 vs 19.66±3.5 p = 0.032) and eGFR (mL/min/1.73m^2^) (6.86±2.46 vs 9.08±4.05 p<0.001) were significantly lower in patients with CKD-MBD compared to non-CKD-MBD (p <0.05) (Table 4). The values of these parameters with survival were assessed and significance (p< 0.01) was seen with levels of urea, BUN, sodium, vitamin D, magnesium, and albumin (Table 4).

**Table 4:** Changes in the levels of the measured parameters with CKD-MBD and survival: BUN: blood urea nitrogen, iCa: ionised calcium, PTH: parathyroid hormone, ALP: alkaline phosphatase, CKD: chronic kidney disease, eGFR: estimated glomerular filtration rate, MHD: maintenance hemodialysis, Ca x P: calcium-phosphate product, CKD-MBD: Chronic Kidney Disease - Mineral Bone Disorder

	CKD-MBD Present	CKD-MBD Absent	p-value	Survival Group	Non-Survival Group	p-value
	Mean ± SD	Mean ± SD		Mean ± SD	Mean ± SD	
Age (years)	48.2±11.65	50.18±11.42	.207	49.61 ± 11.39	51.39 ± 12.78	.476
Urea (mg/dL)	87.22±26.47	73.07±22.6	<0.001	75.18 ± 23.85	89.06 ± 25.33	.008
Creatinine (mg/dL)	8.53±2.39	6.95±2.24	<0.001	7.28 ± 2.33	7.58 ± 2.78	.558
BUN (mg/dL)	40.38±12.25	33.83±10.47	<0.001	34.8 ± 11.04	41.23 ± 11.73	.008
Sodium (mmol/L)	139.46±3.8	139.53±4.05	.896	139.7 ± 3.81	137.22 ± 5.43	.004
Potassium (mmol/L)	4.72±0.67	4.67±0.7	.622	4.68 ± 0.69	4.69 ± 0.7	.966
iCa (mmol/L)	0.96±0.12	1.01±0.12	.002	1 ± 0.12	0.98 ± 0.14	.557
Calcium (mg/dL)	8.61±0.96	9.1±0.73	<0.001	9.02 ± 0.81	8.69 ± 0.83	.061
Ca adjusted by albumin (mg/dL)	8.52±0.93	9.02±0.75	<0.001	8.92 ± 0.81	8.82 ± 0.9	.582
Phosphorous (mg/dL)	5.81±1.4	4.65±1.34	<0.001	4.92 ± 1.44	4.71 ± 1.35	.499
Bicarbonate(mmol/L)	18.61±3.86	19.66±3.5	.032	19.5 ± 3.54	18.56 ± 4.34	.228
PTH (pg/mL)	788.43±485.89	595.79±376.12	.001	642.77 ± 407.01	583.3 ± 452.19	.504
Vitamin D (ng/mL)	41.77±20.36	42.44±20.33	.832	43.25 ± 19.9	26.74 ± 21.08	.004
Iron (ug/dL)	87.79±33.89	90.21±47.03	.700	90.01 ± 44.51	84.92 ± 42.58	.621
Ferritin (ug/L)	925.36±696.03	958.14±717.27	.744	938.32 ± 709.42	1105.62 ± 734.01	.312
Magnesium (mg/dL)	2.52±0.36	2.44±0.37	.189	2.47 ± 0.37	2.17 ± 0.3	.016
Albumin (g/dl)	4.11±0.45	4.1±0.49	.818	4.12 ± 0.45	3.83 ± 0.68	.005
ALP (IU/L)	330.88±217	187.58±160.05	<0.001	220.01 ± 183.14	212.89 ± 197.52	.859
Homocysteine (umol/L)	25.88±23.23	24.11±15.01	.534	24.7 ± 17.45	21.17 ± 14.27	.552
eGFR (mL/min/1.73m^2^)	6.86±2.46	9.08±4.05	<0.001	8.61 ± 3.91	8.33 ± 3.34	.742
Duration of CKD (months)	74.32±48.76	71.84±52.77	.733	73.1 ± 52.03	64.09 ± 49.69	.424
Duration of MHD (months)	58.89±30.81	49.57±34.33	.047	51.95 ± 33.36	47.04 ± 39.12	.504
Ca x P (mg^2^/dL^2^)	50.34±14.08	42.54±13.04	<0.001	44.6 ± 13.72	40.84 ± 12.71	.214

ROC curve was done to assess the role of various markers in the diagnosis of CKD-MBD, it was seen that serum creatinine at a best cut-off of 8.25 mg/dL had a sensitivity of 53% and specificity of 73.4%, phosphorus at a best cut-off of 5.5 mg/dL had a sensitivity of 72.7% and specificity of 81.2%, PTH at a best cut-off of823.7 (pg/mL) had a sensitivity of 50% and specificity of 75.1%, ALP at a best cut-off of 152.5 IU/L had a sensitivity of 87.9% and specificity of 62.4%, the product of calcium and phosphorus at a best cut-off of 48.7 (mg^2^/dL^2^) had a sensitivity of 68.2% and specificity of 72.1% (Table 5).

**Table 5:** Table showing the AUC, best cut-off, sensitivity, and specificity in assessing the CKD-MBD PTH: parathyroid hormone, ALP: alkaline phosphatase, eGFR: estimated glomerular filtration rate, MHD: maintenance hemodialysis, Ca x P: calcium-phosphate product, eGFR: estimated glomerular filtration rate, CKD-MBD: Chronic Kidney Disease - Mineral Bone Disorder

	AUC	p-value	Best cut-off values	Sensitivity	Specificity
Urea (mg/dL)	0.642	<0.001	63.1	84.8	38
Creatinine (mg/dL)	0.676	<0.001	8.25	53	73.4
Calcium (mg/dL)	0.351	<0.001	9.9	13.6	88.2
Phosphorous (mg/dL)	0.731	<0.001	5.5	72.7	81.2
Bicarbonate(mmol/L)	0.422	0.053	27.1	3	99.6
PTH (pg/mL)	0.602	0.012	823.75	50	75.1
ALP (IU/L)	0.762	<0.001	152.5	87.9	62.4
eGFR (mL/min/1.73m^2^)	0.334	<0.001	3.07	100	0.4
Duration of MHD (months)	0.61	0.006	31.5	81.8	40.2
Ca x P (mg^2^/dL^2^)	0.673	<0.001	48.7	68.2	72.1

The survival rate in our cohort (n=312) for a year was calculated. There were 23 deaths and 289 surviving patients. It was observed that mean urea (mg/dL) (89.06 ± 25.33 vs 75.18 ± 23.85, p = 0.008) was significantly higher in the non-survival group whereas mean albumin (g/dL) (3.83 ± 0.68 vs 4.12 ± 0.45, p = 0.005), sodium (mmol/L) (137.22 ± 5.43 vs 139.7 ± 3.81, p = 0.004), magnesium (mg/dL) (2.17 ± 0.3 vs 2.47 ± 0.37, p = 0.016) and vitamin D (ng/dL) (26.74 ± 21.08 vs 43.25 ± 19.9, p = 0.004) were significantly lower in the non-survival group of patients compared to patients who survived p<0.05. Cox regression analysis was carried out to predict the hazard of the presence of the terminal event. A positive relation was observed between serum urea concentrations and survival while a negative relation was seen between serum albumin concentration and survival. An increase in urea concentration predicted mortality in 2.9% of cases and a decreased albumin concentration predicted mortality in 88.1% of cases. We also observed a negative relation between magnesium concentrations and survival which predicted mortality in 90.6% of cases in our study. The biochemical markers associated with survival in diabetes showed significantly lower vitamin D and magnesium while the hypertensive group was associated with decreased levels of vitamin D, bicarbonate, and albumin (Table 6).

**Table 6:** Changes in the levels of biochemical markers in diabetes and HTN with respect to survival: hs-CRP: high-sensitivity C-reactive protein, HTN: hypertension

	Diabetes		p-value	HTN		p-value
	Survival Group	Non-Survival Group		Survival Group	Non-Survival Group	
Bicarbonate (mmol/L)	18.77±3.46	19.15±4.44	0.782	19.75±3.43	17.41±3.94	0.022
Vitamin D (ng/mL)	37.4±17.68	14.26±6.28	0.014	44.82±20.7	27.75±20.11	0.027
Magnesium (mg/dL)	2.51±0.35	2±0.1	0.018	2.47±0.38	2.26±0.38	0.240
Albumin (g/dL)	4±0.46	3.83±0.76	0.370	4.18±0.45	3.84±0.62	0.013
hs-CRP (mg/dL)	1.12±1.24	4.39±5.45	0.005	1.19±2.05	1.08±0.77	0.905

## Discussion

Biochemical markers are representations of any disease pathology that help in diagnosis and assessing prognosis. This is valid for conditions like CKD and ESRD, where clinical judgment is primarily based on the measurement of these analytes. CKD grades 4 and 5 with an eGFR of less than 30 mL/min/1.73 m^2^ and less than 15 mL/min/1.73 m^2^, respectively, are generally grouped as ESRD, a single entity regardless of its aetiology, which is an irreversible state of kidney damage requiring renal replacement therapy to maintain life, slow the progression of the disease, and help in reducing complications. Although deteriorating kidney function is a common pathology, there is a need to consider the pathophysiology of the underlying disease, as it is known to affect the nephron differently.

Alterations in renal function affect many different biochemical analytes due to reduced production by the kidneys, reduced clearance, or altered physiology in the patients. Biochemical evaluation of the patient is a prerequisite to RRT therapy and their levels determine the degree of intervention and the type of dialysis. Newer markers of kidney function, although identified, have not found applicability in routine analysis due to their limited availability, lack of standardization, and limited research. With an increasing number of patients on maintenance hemodialysis, the evaluation and management of these patients using routine parameters or a combination of the parameters becomes critical.

In the present study, we found that patients with MHD with a disease duration of more than 62 months (5 years) had a significant increase in the serum levels of mean ALP, vitamin D, and iron levels with significantly high correlations. The duration of the kidney disease, regardless of the aetiology, causes loss of renal function. ALP is a traditional marker of bone function that reflects changes in calcium, phosphorus, and other bone markers. It also inactivates pyrophosphate, an endogenous inhibitor of hydroxyapatite formation. In CKD, vascular cells undergo osteoblastic differentiation and express several bone-associated proteins, including ALP. Increased ALP levels have been independently associated with arterial stiffening and vascular calcification, thereby contributing to cardiovascular disease and mortality in CKD.

The increase in levels of vitamin D and iron in patients with a longer history of CKD duration can be attributed to the replacement therapy that is initiated in these patients to prevent or correct anaemia and the development of bone disease. Serum iron levels, though normally used as indicators of anaemia, are also known to increase oxidative stress and add to the chronic inflammation present in renal disease. What has to be kept in mind during replacement therapy in patients with MHD is that significant increases in serum iron levels due to overzealous treatments may contribute to the inflammatory milieu and result in higher mortality [9]. 

We next grouped our patients based on the duration of haemodialysis to understand the changes in the markers that were altered with the treatment. We found that there was a significant increase in serum levels of PTH, vitamin D, ferritin, and ALP, and an inverse relationship between magnesium and homocysteine was observed. 

Hypocalcemia, hyperphosphatemia, and elevated parathyroid hormone levels may additionally occur as a result of renal failure [ 12, 13]. Hypocalcemia occurs due to decreased production of active vitamin D (1,25 dihydroxyvitamin D), which is responsible for gastrointestinal (GI) absorption of calcium and phosphorus and suppression of parathyroid hormone excretion. Hyperphosphatemia occurs because of impaired phosphate excretion in the setting of renal failure [14, 15 ]. Both hypocalcemia and hyperphosphatemia stimulate hypertrophy of the parathyroid gland and result in increased production and secretion of parathyroid hormone. Altogether, these changes in calcium metabolism can result in osteodystrophy (renal bone disease) and may lead to calcium deposition throughout the body (i.e., metastatic calcification).

Magnesium is the second most important intracellular cation and is required as a cofactor in multiple enzymatic reactions and metabolisms [11]. It is important in regulating mineral bone metabolism, adenosine triphosphate metabolism, neurotransmitter release, and regulation of vascular tone, heart rhythm, neuromuscular processes, and platelet-activated thrombosis [16]. Magnesium possesses an anti-atherosclerotic effect, which is mediated partly via its anti-inflammatory and antioxidant properties; conversely, by inhibiting endothelial proliferation, upregulating plasminogen activator inhibitor-1 and vascular cell adhesion molecule-1, magnesium deficiency promotes endothelial dysfunction [17, 18].

A growing body of literature associates both hypomagnesaemia as well as hypermagnesaemia with important clinical endpoints such as an increased risk of cardiovascular disease (CVD), arrhythmias, and mortality. Previous studies have shown that low magnesium levels are associated with CVD, ischaemic heart disease, endothelial dysfunction, and atherosclerosis. A magnesium deficiency promotes hydroxyapatite formation and calcification of vascular smooth muscle cells and is closely related to increased insulin resistance and metabolic syndrome [19, 20]. Hypomagnesaemia is also associated with hypokalemia, knowing the results of magnesium, a tailor-made dialysate magnesium should be implemented in daily clinical practices.

In a recently published review, the authors recommend that magnesium should be measured regularly and adjust dialysate magnesium accordingly to maintain plasma magnesium within the normal range [21]. An understanding of the physiology of magnesium homeostasis and handling is of prime relevance for those taking care of patients with CKD and ESRD. Besides magnesium, we also found that duration was found to be significantly positively increased with the duration of dialysis. In contrast, homocysteine levels were increased with shorter duration, indicating that the increase in this marker is more related to the acute presentations rather than in the long-standing condition.

A significant increase in the levels of serum ferritin was noted in patients with a higher duration of dialysis. It has to be kept in mind that serum ferritin is also an acute phase reactant and can be increased in states of malnutrition and inflammation. When increased, it can cause damage to the kidney and create a prooxidant state that is detrimental in patients with ESRD. A judicious iron replacement protocol thus has to be followed to balance the pros and cons of treatment [22].

We next grouped the patients undergoing MHD based on the etiology of CKD. Patients having ESRD due to systemic disease showed a significant increase in the levels of mean urea and potassium when compared to those with non-systemic diseases (Table 2). Systemic diseases, diabetes, and hypertension are known to cause distinct pathological changes within the kidney architecture altering its function that leads to increased blood levels of urea and potassium [23]. Urea serves not only as a means for excreting nitrogen but also plays a central role in controlling the flow rate in the collecting ducts and in concentrating the urine by absorption and reabsorption process via specific urea transport proteins. A decrease in excretion of urea potentially lowers the flow rate in the terminal cortical collecting duct causing a diminished rate of excretion of potassium and hyperkalemia [24].

Serum levels of potassium, in turn, are directly responsible for regulating the number of urea transporters, and a depletion of potassium has been shown to reduce the abundance of urea transporters in the renal medulla in mice, leading to uremia and a simultaneous increase in blood urea nitrogen [23]. In addition to these alterations, an increase in the amount of toxic metabolites and reactive oxygen species noted in uremic patients causes or worsens insulin resistance through uremia-induced toxicity and alterations of complex metabolic pathways [25,26]. Many of these alterations are considered to be due to post-translational modifications and carbamylation of different proteins, which disrupt their structure and interfere with signalling pathways. Our findings are in line with Stim et al., who reported an increase in BUN in patients with ESRD with diabetes compared to normal individuals. This increase in BUN paralleled an increase in carbamylation of proteins such as haemoglobin and albumin [27, 28]. 

We next compared the MHD patients with known inciting systemic diseases, namely diabetes and hypertension. The patients identified with a previous history of hypertension belonged to a significantly lower age group with a higher CKD and MHD duration in comparison to those patients with diabetes. We observed a significant increase in serum levels of albumin, creatinine, and vitamin D in comparison to the diabetic group, while eGFR was significantly reduced (Table 3). 

Urinary albumin is the most abundant protein found in urine. Albuminuria may precede the decline in kidney function and is associated with disease progression and poor outcomes. Increased level of serum albumin in the hypertensive group is attributed to the increased proteinuria, or more specifically, albuminuria, due to pathological changes and damage to the glomerulus rather than to the tubulointerstitial apparatus of the nephron. Increased albumin in the urine is indicative of glomerular proteinuria, whereas tubular and overflow proteinuria cannot be characterized by albuminuria alone [14]. The diabetic group, on the other hand, showed high hs-CRP, indicating the presence of an ongoing chronic inflammatory pathology.

**Figure 1:**
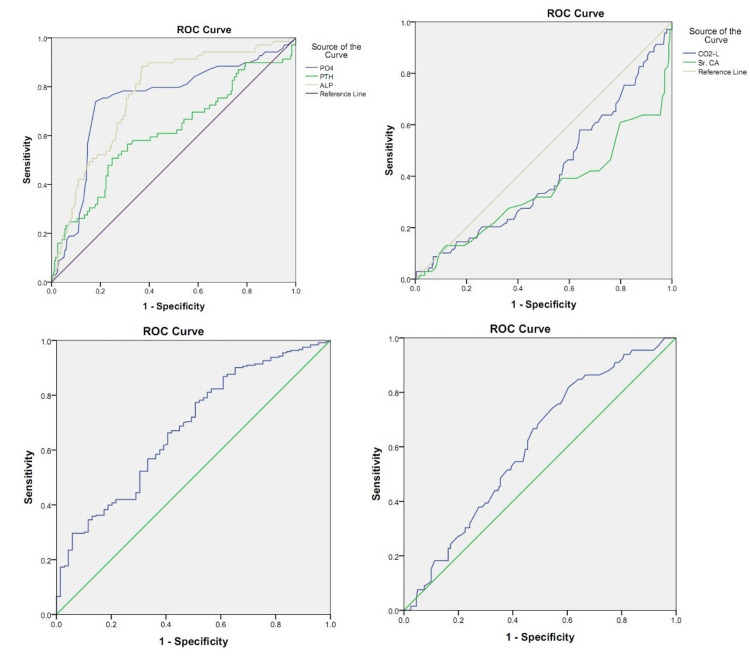
ROC curves showing the significant markers in the different groups of patients on MHD ALP: alkaline Phosphatase, PO_4_: phosphorus, PTH: parathyroid hormone, SR CA: serum calcium, CO_2_-L: bicarbonate, ROC: receiver operating characteristic, MHD: maintenance hemodialysis

CKD-MBD is an important systemic complication of CKD that evolves with the worsening of the disease and eGFR and requires an early diagnosis to mitigate its progress. This places emphasis on stringent laboratory monitoring of PTH, ALP, calcium, phosphorus, bicarbonate, and vitamin D levels. Kidney Disease Improving Global Outcomes (KDIGO) guidelines suggest measurements of serum PTH or specific ALP to evaluate bone disease as predictors of underlying bone turnover [29]. In our study, 69 patients (22%) were found to have increased levels of urea, creatinine, PTH, ALP, phosphorus, product of calcium and phosphorus, and vitamin D levels indicating the presence of CKD-MBD (Table 4) [15]. The levels of mean ionized calcium, serum calcium, corrected calcium, bicarbonate, and eGFR, on the other hand, were significantly lower. Increased levels of urea and creatinine are clinical indicators of worsening renal function. The area under the curve (AUC), best cut-off, sensitivity, and specificity using these markers in assessing the CKD-MBD were determined in the present study (Table 5, Figure 1).

Increased urea in itself causes imbalances in fluid, electrolyte, and hormonal balances, metabolic abnormalities, and a buildup of uremic toxins, which in turn leads to abnormalities in cardiac function. PTH, ALP, and calcium together participate in the complex regulation of calcium and phosphorus homeostasis through the relationship between the bones, intestine, kidneys, and parathyroid glands. 

Hyperparathyroidism secondary to renal disease, termed renal hyperparathyroidism, is a common complication of CKD along with deranged calcium, phosphorus, and vitamin D homeostasis. It is characterized by a complicated, multifaceted, and as yet incompletely understood pathophysiology. It is estimated that 30%-50% of stage 5 CKD patients have PTH levels of >300 pg/mL [30]. Elevated PTH levels occur in response to hypocalcemia induced by phosphate retention and reduced calcitriol synthesis as a consequence of reduced renal function [31].

In our present study, we found a significant increase in the levels of PTH that were significantly correlated with increased ALP and decreased serum calcium levels. No significant correlation between the PTH and vitamin D was noted in our study, as the patients received vitamin D replacements as part of therapy. Correction of 25-hydroxyvitamin D deficiency can partially correct elevated PTH levels in patients with mild to severe CKD. Beyond its role in bone turnover, elevated PTH, in the long run, mediates vascular and valvular calcification, cardiovascular fibrosis, and apoptosis through the transforming growth factor beta (TGF-β) signalling pathway. Persistent high levels of PTH are also considered similar to the uremic toxin due to its similar presentation and adverse effects on the cellular function of different target organs.

The Dialysis Outcomes and Practice Patterns Study (DOPPS) has denoted that serum PTH higher than 600 pg/mL (63 pmol/L) is associated with a 21% increase in all-cause mortality risk [32]. ALP is a dephosphorylating hydrolyzing enzyme operating in an alkaline environment with a wide distribution (bone, liver, placenta, leukocytes, and kidneys). Elevated levels are commonly seen in diseases with a high bone turnover and in cases of CKD-MBD. However, ALP may be more than a mere marker of bone turnover. Previous studies have found that ALP is causally involved in the cardiovascular calcification of CKD, with higher mortality, and likely represents a less biased tool than PTH to assess the risk(s) imparted by CKD-MBD [33]. Higher ALP has been shown to result in increased hydrolysis of pyrophosphate, a potent inhibitor of vascular calcification and lower levels are shown to lower smooth muscle calcification in animal models and are associated with a linear decrease in mortality [34].

Elevations in phosphate levels compromise the vasculature, resulting in ischemic changes with a poor prognosis [35]. We also found a significant increase in the levels of serum phosphorus in the patients with CKD-MBD in comparison to those without. Similar to ALP, serum phosphorus has also been found to be an independent risk factor for a more rapid decline in renal function and a cause of higher mortality. Hyperphosphatemia is known to produce precipitation and deposition of calcium phosphorus microcrystals in the renal tubular lumen, capillaries, and interstitium resulting in progressive functional deterioration in chronic renal failure. Animal models of CKD have shown that calcium-phosphorous crystals evoke an inflammatory response leading to fibrosis, loss of nephrons, and eventually to chronic renal failure, while in a uremic rat model, high dietary phosphorus levels and hyperphosphatemia both induced cardiac fibrosis and arterial wall thickening by ossification of vascular smooth muscle cells [36].

Elevations in serum PTH, urea, ALP, phosphorous, and calcium-phosphate products together are intimately involved in promoting calcification. Patients with ESRD have a higher prevalence of vascular calcification than the general population, and 80-90% of patients with ESRD have been reported to have some degree of vascular calcification [37]. An additional risk of CKD-related bone loss is its association with extraskeletal calcification, which contributes to excessive CVD morbidity and mortality of CKD. An elevated PTH level, along with increasing ALP and urea in conjunction, would serve as markers for bone disease and determinants for developing vascular calcifications, avoiding the use of expensive testing modalities [38]. An increase in these levels, along with increasing calcium, should be considered to increase this risk, and any therapeutic administration of vitamin D and calcium in this setting should be used with caution. Utilizing the differences in levels of these multiple-biomarkers can help to identify the aetiology, prognosis, and risk profile for patients with MHD.

In the present study, the survival of the patients was assessed and equalled 92.7% at the end of the study period or censored at the time of death. Our mortality rate (7.3%) was slightly higher when compared to Chandrashekhar et al [39]. Increased serum levels of urea and decreased levels of serum albumin, sodium, vitamin D, and magnesium were significantly associated with non-survival. Previous studies have demonstrated uremia as a poor prognostic marker that predisposes patients to develop adverse cardiac events, spontaneous subdural hematomas, uremic encephalopathy, or stroke. Our findings of uraemia and hypoalbuminaemia, less than 4 g/dL, associated with higher mortality are in line with the findings of Dwyer et al. and Owen et al. [40,41].

The observed decrease in sodium and magnesium levels with an increase in mortality in our study is also corroborated with the findings of Lacson et al., who showed that increased serum magnesium levels reduce the relative risk of mortality in a large cohort of haemodialysis patients [42]. Mortality in diabetics was associated with significantly lower vitamin D and magnesium levels. In contrast, in the hypertensive group, it was associated with decreased levels of vitamin D, bicarbonate, and albumin levels (Table 6). Judicious concerted use of these markers in evaluation and follow-up will serve as indicators of survival, worsening of disease, or as prognostic markers.

The findings in our study of alterations in biochemical markers of renal disease can be used to design panels and assign scores. These scores can help in assessing the risk or the prognosis of the patients without using expensive tests that will add to the already burdened dialysis patient. The markers of renal function used in routine clinical practice can serve as more effective tools when used in conjunction with the others. 

Limitations

The study was limited to a single centre and a particular patient population. The multicentric study would help in better generalizability of findings. The study duration was only one year, which may act as a limitation in tracking long-term patterns and trends in CKD patients. The study has not taken into account the efficacy of various treatments. There was a single point of measurement of laboratory parameters right before a dialysis session. The repeated measurements throughout time can help in better understanding the intra-patient variability.

## Conclusions

The markers of renal function used in routine clinical practice can serve as more effective tools when used in conjunction with the others. The increased levels of PTH in conjunction with ALP not only indicate bone disease but can also reflect the presence of vascular calcifications, while decreased serum magnesium levels along with albumin can help in predicting the survival of the patients. Our data provide an important contribution to understanding the different parameters that are changed with the difference in the duration of kidney disease, the duration of haemodialysis, and the presence or absence of systemic disease. Using these markers as scores can improve management and guide risk assessment for patients with MHD. 
